# Soluble Activin Receptor Type IIB Improves Muscle Regeneration Following *Crotalus atrox* Venom-Induced Damage

**DOI:** 10.3390/toxins17020059

**Published:** 2025-01-28

**Authors:** Medha Sonavane, Ali Alqallaf, Robert D. Mitchell, José R. Almeida, Soheil Gilabadi, Nicholas J. Richards, Sodiq Adeyemi, Jarred Williams, Olli Ritvos, Sakthivel Vaiyapuri, Ketan Patel

**Affiliations:** 1 School of Pharmacy, University of Reading, Reading RG6 6UB, UK; medhasonavane87@gmail.com (M.S.); j.r.dealmeida@reading.ac.uk (J.R.A.); s.gilabadi@pgr.reading.ac.uk (S.G.); j.williams4@pgr.reading.ac.uk (J.W.); 2 School of Biological Sciences, University of Reading, Reading RG6 6UB, UK; dr.a.alqallaf@gmail.com (A.A.); n.j.richards@pgr.reading.ac.uk (N.J.R.); s.o.adeyemi@pgr.reading.ac.uk (S.A.); 3 Medical Services Authority, Ministry of Defence, Kuwait City 13012, Kuwait; 4 Micregen Ltd., Thames Valley Science Park, Reading RG2 9LH, UK; robertmitchell@micregen.com; 5 Department of Physiology, Medicum, Faculty of Medicine, University of Helsinki, 00014 Helsinki, Finland; olli.ritvos@helsinki.fi

**Keywords:** soluble activin receptor type IIB, *Crotalus atrox*, venom, myonecrosis, fibrosis and muscle regeneration

## Abstract

Viper bite envenoming often results in prominent skeletal muscle damage. According to our previous studies, the prolonged presence of *Crotalus atrox* venom toxins induced extensive muscle damage, which mimicked the outcome of chronic muscle damage often seen in human muscular dystrophies. In the case of chronic muscle damage, two critical processes occur: muscle regeneration is impaired, and fibrosis develops. Myostatin/activin signalling is a key regulator of both of these processes. Myostatin and its closely related molecules, in particular activin, inhibit the proliferation and differentiation of myocytes while promoting proliferation of fibroblasts and expression of extracellular matrix proteins. Thus, attenuating myostatin/activin signalling offers an attractive means of promoting muscle development while decreasing fibrosis. Hence, we have used the soluble activin receptor type IIb, which acts as a ligand trap for both myostatin and activin, to dampen signalling and assessed whether this intervention could alter the pathological trajectory of *C. atrox* venom-induced muscle damage in mice. We report that the soluble activin receptor type IIb treatment increased the size of regenerating fibres while reducing the level of fibrotic tissues in venom-damaged muscle.

## 1. Introduction

Muscle damage is one of the most severe consequences of snakebite envenoming (SBE), specifically following viper bites [1,2]. The annual global burden of SBE-mediated disabilities is around 400,000 [3]. In the US, around 9000 people are affected by snakebites every year [4]. The Western Diamondback rattlesnake (*Crotalus atrox*) is prominent in southwestern USA and Mexico and is responsible for most SBE-induced fatalities in northern Mexico [5]. Viper snakes are primarily responsible for SBE-mediated skeletal muscle damage with permanent consequences for the victims [6,7]. Despite this, treatment options for SBE-induced muscle damage are limited [8].

Snake venoms contain numerous enzymatic and non-enzymatic proteins with myotoxic and haemotoxic properties [9,10]. In fact, due to the abundance of components targeting muscle, snake venoms serve as a widely recognised research tool to investigate the nature and dynamics of muscle regeneration [11]. For instance, cardiotoxin-mediated injury has contributed to many discoveries and significant advancements in our knowledge of muscle physiology and related pathologies [12]. The action of this single purified snake venom toxin provided an ideal model that created the foundation for current insights into muscle regeneration and is crucial for the evaluation and development of novel therapies [13]. In contrast, snake venoms exhibit greater complexity, affecting multiple targets with different molecular mechanisms [14]. Other snake venom toxins, such as phospholipase A_2_ (PLA_2_) and metalloproteases (svMPs), operate through membranolytic or proteolytic mechanisms, respectively, causing significant muscle necrosis, by disrupting the cell membrane or altering the structure and function of the extracellular matrix (ECM) components [15,16]. The synergistic, localised, and fast interaction of venom toxins with cellular components challenges traditionally used immunoglobulin-based therapy and often leads to poor muscle regeneration [17,18,19].

In clinical settings, tissue damage, necrosis, and muscle fibrosis are usually treated with surgical procedures such as debridement, fasciotomy, or amputations [17,20,21]. From both compositional and functional perspectives, *C. atrox* venom comprises a biochemical cocktail, including ssvMPs, snake venom serine proteases (svSPs), PLA_2_, L-amino acid oxidase (LAAO), and C-type lectin-like proteins [22]. These components contribute to haemodynamic disturbances and extensive muscle damage [22,23]. We have previously shown that one component from *C. atrox* venom termed CAMP (*Crotalus atrox* metalloprotease) is capable of inducing permanent muscle damage when injected into the tibialis anterior (TA) muscle of mice [24]. Our investigations showed that this toxin persists for days in the damaged muscle and suggested that it continually interferes with innate muscle regeneration, which ultimately dysregulates muscle growth [25]. The resultant outcome of continued degeneration/regeneration mimics chronic muscle damage often seen in a spectrum of human muscle diseases, including Duchenne Muscular Dystrophy, which manifests in the formation of fibrotic tissues and poorly developed myofibres [24]. In this context, utilising whole venom instead of cardiotoxin-induced injury models allows for a more realistic simulation of the combined effects of various tissue-damaging toxins, resulting in fibrosis and functional impairments, consistent with the detrimental consequences observed in SBE victims [17,26,27,28].

The whole process of muscle regeneration is underpinned by the presence of a resident population of stem cells called ‘satellite cells’ (SC) [29]. Nevertheless, several other key factors are required for muscle regeneration. The entire process can be divided into two components: the clearance of damaged tissues and the reconstitution of lost fibres [30]. Acute muscle damage results in the infiltration of neutrophils and activation of monocytes, which develop an ‘inflammatory environment’ that not only clears cell debris but also promotes the activation of two important cell types: SC and fibro-adipogenic progenitors (FAPs) [31]. FAPs act to promote expansion and self-renewal of SC, as well as remodelling the ECM, key steps towards muscle regeneration [32,33]. Importantly, the proinflammatory environment controls the development of FAPs to maintain them as precursors, as well as limiting their number through the induction of apoptosis [34]. As the regeneration process continues, the environment becomes anti-inflammatory due to the activity of M2 macrophages [35,36]. These cells secrete various factors that promote differentiation of SC to reconstitute lost myofibres but can also lead to the differentiation of any active FAPs into either fibroblasts and/or adipocytes [37]. In normal regeneration, SC numbers are expanded, and they differentiate into myoblasts, which then fuse to form myofibres. The FAPs are then eliminated, resulting in the absence of fibrosis or fat deposition. However, in chronic muscle damage, the different phases of regeneration become entangled so that they are no longer occurring in an orderly sequence; rather, they might be happening simultaneously and so interfere with each other [38]. Two consequences of this degeneration process are the differentiation of FAPs into fibroblasts as well as poorly developed myofibres [34].

Signalling mediated by myostatin and its closely related family members, especially activin, is important for muscle regeneration [39]. Myostatin and activin, members of the transforming growth factor (TGF) β-superfamily of secreted proteins, signal by binding to the extracellular portion of the activin type II receptor [40]. This leads to a series of intracellular events mediated by a signalling protein, Smad2/3. In muscle cells, this signalling inhibits proliferation and differentiation of myoblasts, and in muscle fibres it leads to decreased protein synthesis [41,42]. However, it promotes fibroblast proliferation, which then can differentiate into myofibroblasts, which are responsible for the formation of fibrotic tissues [43]. Hence, the inhibition of myostatin/activin signalling has become an attractive proposition to overcome some of the issues associated with chronic muscle damage (poor muscle formation and fibrosis). Numerous strategies have been developed to attenuate myostatin/activin signalling [44,45]. We have developed a soluble ligand trap, based on the extracellular portion of the activin type IIB receptor (sActRIIB), which acts to sequester myostatin and activin, thus rendering them incapable of binding to their normal receptor [46]. In this study, we demonstrate that sActRIIB treatment following *C. atrox* venom-induced muscle damage promotes muscle regeneration and decreases fibrosis.

## 2. Results

### 2.1. sActRIIB Treatment Increases the Weight of C. atrox Venom-Damaged TA Muscles

**Figure 1:**
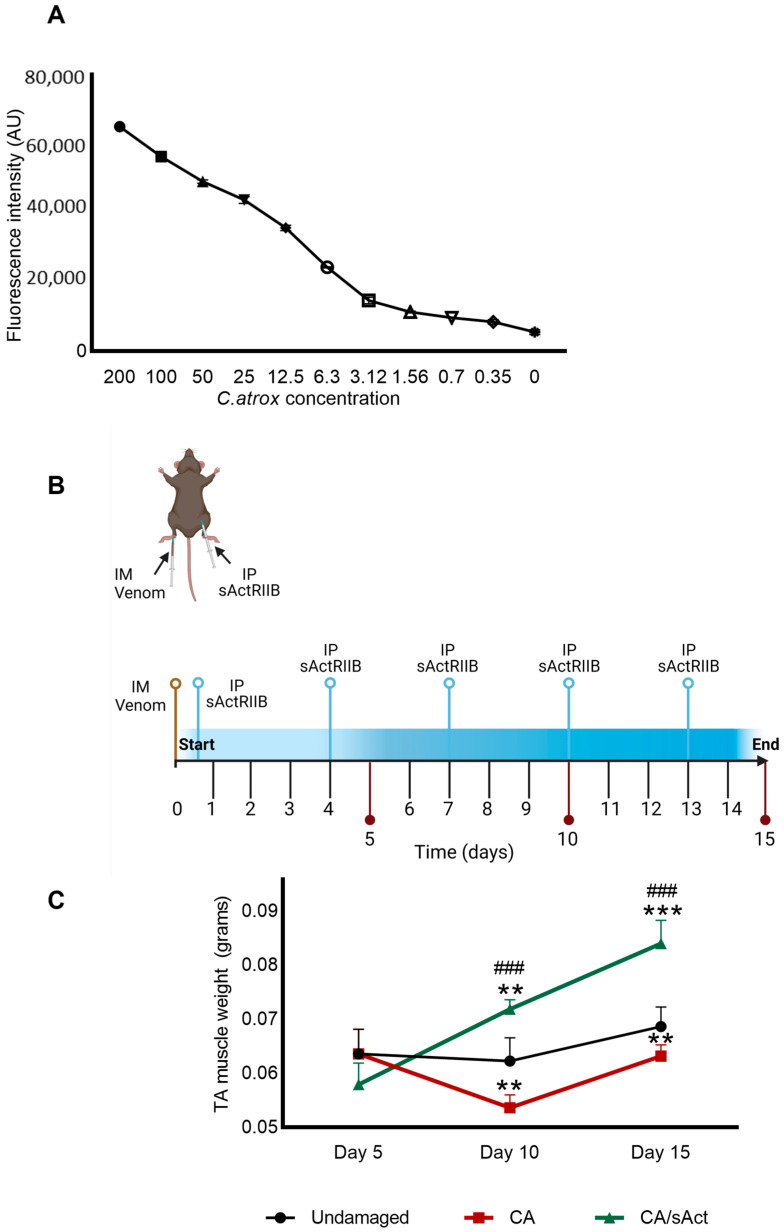
**Metalloprotease activity of *C. atrox* venom and its impact on TA muscle in mice.** (**A**) Quantification of metalloprotease activity at differing concentrations of *C. atrox* venom (µg/mL) using DQ^TM^-gelatin as a substrate. (**B**) Graphical representation of in vivo muscle damage protocol. Following injection of *C. atrox* venom into the TA muscle, mice were administered intraperitoneally with sActRIIB at one hour after the venom injection and thereafter every third day. Mice were sacrificed at either day 5, 10, or 15. This image was created using BioRender. (**C**) TA muscle weights in the three cohorts of mice over time. Data represent mean ± S.D. (*n* = 4 mice per cohort). * represents the comparison between undamaged muscle with either CA or CA/sAct cohorts, and # symbol represents the comparison between CA and CA/sAct cohorts at the same time point. Statistical analysis was performed using one-way ANOVA followed by Tukey’s post-hoc test. ** *p* < 0.01 and *** or ### *p* < 0.001.

Degradation of the ECM is a key step in venom-induced skeletal muscle damage [47,48]. Hence, we conducted in vitro experiments to quantify collagenolytic activity of whole *C. atrox* venom with the specific aim of determining the concentration of venom that could be deployed in vivo. Using DQ^TM^-gelatin, we found that *C. atrox* venom showed high levels of collagenolytic activity (Figure 1A). Using this data, together with our previous studies, we identified that a dose of 0.25 μg/g of body weight would cause significant muscle damage in a mouse model.

Thereafter, we first aimed to determine the impact of *C. atrox* venom when injected into a mouse TA muscle. Secondly, we assessed the effect of sActRIIB on the muscle regeneration process following *C. atrox* venom-induced damage (Figure 1B). To that end, we developed two cohorts of mice. The first group was treated with *C. atrox* venom injected into the TA muscle (CA muscle group), whereas the second group had both a TA injection of *C. atrox* venom and an intraperitoneal injection of sActRIIB treatment at different time points (CA/sAct muscle group) (Figure 1B). The contralateral muscle from the CA group was used as an undamaged control muscle (UD muscle group). Muscles were collected at different time points and weighed to determine the overall impact of the treatment. On day 5, there were no significant differences in TA weights between the three cohorts (Figure 1C). However, on day 10, CA muscles showed a significant decrease in weight compared to UD, whereas CA/sAct showed a significant increase compared to UD. At this timepoint, the TA muscles from CA/sAct group were significantly heavier than those of CA mice. On day 15, CA muscles were still significantly lighter than those of UD, whereas CA/sAct muscles showed significant weight increases compared to the other two groups (Figure 1C).

### 2.2. sActRIIB Treatment Promotes Muscle Regeneration and Attenuates Fibrosis

**Figure 2:**
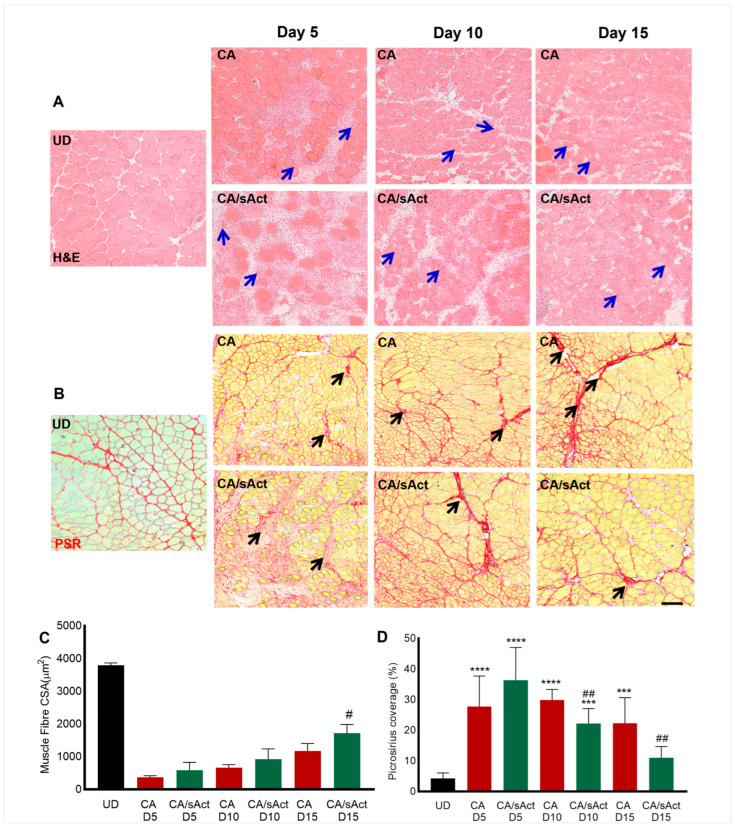
**Histological analysis of muscle regeneration and fibrosis.** (**A**) H&E staining for TA muscle sections on UD, CA and CA with sActRIIB on days 5, 10, and 15. Blue arrows show fibres with CLN. (**B**) Picrosirius red staining for TA muscle sections on days 5, 10, and 15. The black arrows show fibrotic regions. (**C**) Area of muscle fibres with CLN at different time points. (**D**) Quantification of picrosirius red staining area in the damaged region. Data represent mean ± S.D. (*n* = 4 mice per cohort). * represents a comparison between UD muscle with either CA or CA/sAct groups. # represents a comparison between CA and CA/sAct cohorts at identical times post-venom injection. The data were quantified from a minimum of 30 measures from each mouse. One-way ANOVA followed by Tukey’s post-hoc test was used to calculate the statistical significance. # *p* < 0.05, ## *p* < 0.01, *** *p* < 0.001 or **** *p* < 0.0001. The scale bar for all images represents 100 μm.

The changes in muscle weights described above could simply be due to oedema induced by muscle damage. In order to determine the cause of the change in muscle weights, we conducted a thorough histological profiling of the samples. Muscle sections did not show gross features of oedema. Hence, we firstly investigated the presence of muscle fibres with centrally located nuclei (CLN), a marker for newly formed muscle fibres. As expected, there were no such fibres with CLN in the UD TA muscle in H&E-stained sections (Figure 2A). We therefore compared the size of muscle fibres displaying CLN between the CA and CA/sAct groups at the same time points. The results showed that newly formed fibres were larger in the CA/sAct cohort compared to CA on day 15 (Figure 2A,C). Interestingly, even though the sActRIIB induced fibre enlargement, they were still smaller than UD fibres (black bar in Figure 2C). Infiltrating cells were present at all time points in both cohorts, which decreased in number over time (Supplementary Figure S1).

Next, we analysed whether sActRIIB played any role in modifying the development of fibrotic tissues after *C. atrox* venom injection. The degree of picrosirius red staining was used to visualise collagen (at early time points) or total fibrosis (at later time points). Picrosirius red staining was present in UD muscle, which marks the connective tissue in healthy muscle, which accounts for approximately 4% of the area (Figure 2B,D). Significantly elevated levels of picrosirius red staining were observed in all muscle samples compared to the UD at all time points from both experimental cohorts, the exception being the CA/sAct muscle on day 15 (Figure 2B,D). When comparing between CA and CA/sAct at same time point, there was significantly lower picrosirius staining in the CA/sAct muscle on days 10 and 15. Hence, we observed that with persistent dosing of sActRIIB, venom-induced muscle fibrosis was significantly reduced. Hence, the enlargement of newly generated fibres and decrease in fibrosis was induced by sActRIIB treatment at later time points.

### 2.3. sActRIIB Treatment Reduced Tissue Necrosis but Did Not Affect the Early Stages of Muscle Regeneration

**Figure 3:**
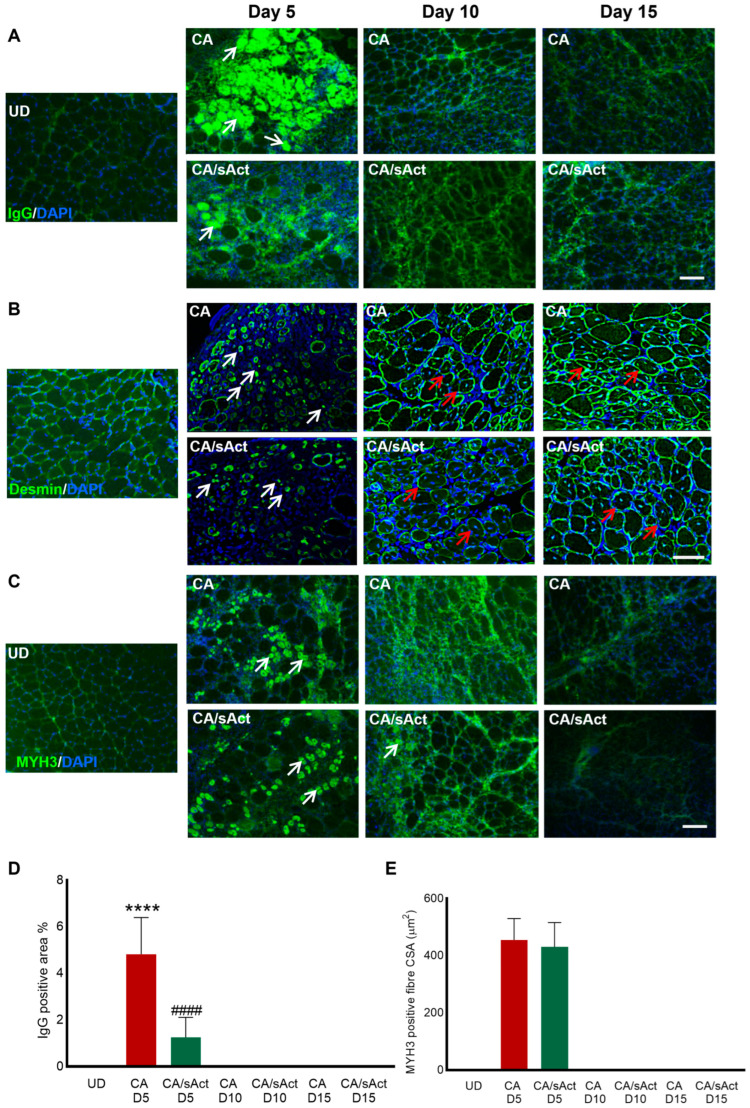
**Profiling *C. atrox* venom-induced degeneration and regeneration.** (**A**) IgG infiltration in the muscle fibres in UD, CA, and CA/sAct muscle on days 5, 10, and 15. White arrows indicate infiltrated fibres. (**B**) The expression of desmin was analysed in different muscle sections using immunohistochemistry. White arrows show expression of desmin in myoblasts. Mature fibres show expression of desmin at the edge of the fibres (indicated using red arrows). (**C**) Myofibres expressing MYH3. White arrows indicate newly formed fibres. (**D**) Quantification of IgG-positive fibres (% in 200 μm × 200 μm area). (**E**) Quantification of fibre size showing MYH3 expression. Data represent mean ± S.D. (*n* = 4 mice per cohort). The * symbol represents a comparison between UD muscle with either CA or CA/sAct, and # symbol represents a comparison between CA and CA/sAct at the same time point. The data were obtained from a minimum of 30 measures from each mouse. Statistical analysis was performed using one-way ANOVA. **** or #### *p* < 0.0001. The scale bar for all images represents 100 μm.

Viper venom is known to induce muscle necrosis and attenuate regeneration [49]. Dying/necrotising fibres facilitate the infiltration of circulating immunoglobulins (IgG), activation of regeneration through the development of myoblasts, and, finally, the formation of newly regenerating fibres expressing an embryonic myosin heavy chain (MYH3) [50]. Hence, we analysed muscle necrosis (IgG) and the formation of new fibres (desmin, and MYH3) by staining for relevant markers. The UD samples had no muscle fibres infiltrated with IgG (Figure 3A,D). However, CA group showed a substantial infiltration of IgG representing significant necrosis. Examination of the necrotic fibre profile in the treated cohorts showed approximately a 50% reduction with sActRIIB treatment on day 5. Thereafter, no evidence of necrosis was detected across all the cohorts (Figure 3A,D). Next, we examined the early stages of fibre development. Firstly, we examined the expression of desmin, as this protein is firstly expressed in myoblasts, where it is located throughout the cytoplasm before being concentrated at the edge of myofibres [51]. The profiling showed approximately the same density of cells expressing desmin in the cytoplasm (average 14 in a 200 μm × 200 μm area) of both the untreated and treated muscles on day 5 (Figure 3B). Thereafter, cytoplasmic expression of desmin was rarely found in sections from either cohort. We then examined the expression of another marker of regeneration, MHY3. There were no MYH3-positive fibres in the UD samples (Figure 3C). CA muscles had MYH3 expression only on day 5. CA/sAct muscles also had the same level of MYH3 expression on day 5. However, treatment with sActRIIB did not alter the size of the regenerating fibres expressing MYH3 (Figure 3C,E). We did identify a very low number of MYH3 (1-3)-positive fibres (at a lesser intensity compared to day 5) in 2/4 CA/sAct muscles on day 10. However, as these were so infrequent, albeit at a relative size comparable to day 5, we have omitted them from Figure 3E. Therefore, sActRIIB acts to modify the degeneration programme, evidenced by the number of fibres being affected in the first instance but not the early stages of muscle fibre regeneration, as the number of desmin-positive myoblasts and size of MYH3-expressing fibres was similar in both cohorts.

### 2.4. sActRIIB Treatment Promoted the Remodelling of the ECM

**Figure 4:**
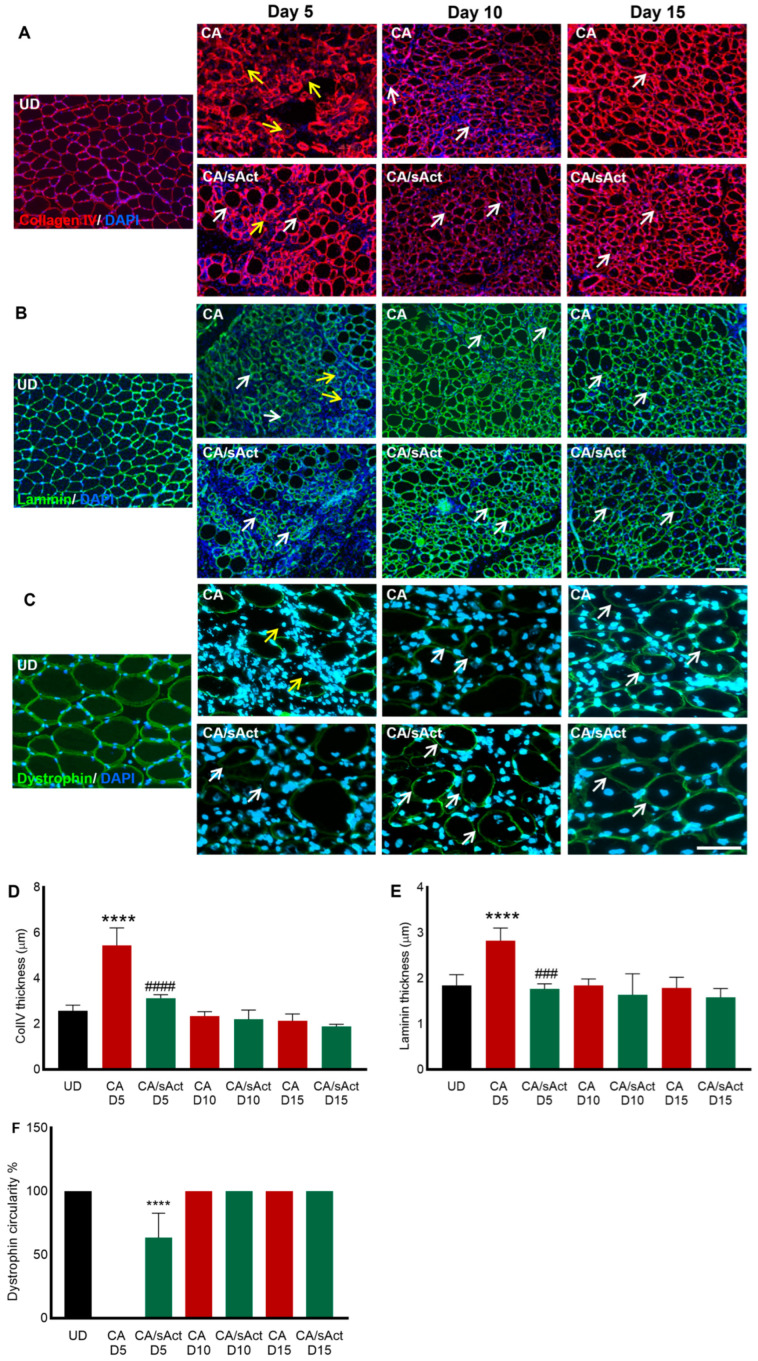
**The organisation of the ECM and dystrophin expression in regenerating fibres.** (**A**) Collagen IV in the muscle fibres in UD, CA, and CA/sAct muscle on days 5, 10, and 15. Yellow arrows indicate uneven collagen IV expression around fibres, and white arrows show even expression of this protein. (**B**) Laminin expression in muscle sections at various time points. Yellow arrows indicate uneven laminin expression around fibres, and white arrows show evenly expresseiprotein. (**C**) Dystrophin expression around regenerating fibres. Yellow arrows indicate regenerating (presence of CLN) fibres lacking dystrophin expression, and white arrows show regenerating (presence of CLN) fibres with dystrophin around the entire fibre. Quantification of collagen IV thickness (**D**), laminin (**E**) and dystrophin circularity (**F**) was performed using ImageJ. Data represent mean ± S.D. (*n* = 4 mice in each cohort). * represents a comparison between UD muscle with either CA or CA/sAct, and # represents a comparison between CA and CA/sAct cohorts at identical times. The data were obtained from a minimum of 30 measures from each mouse. Statistical analysis was performed using one-way ANOVA followed by Tukey’s post-hoc test. ### *p* < 0.001 and **** or #### *p* < 0.0001. The scale bar in (**A**,**B**) represents 100 μm. The scale bar in (**C**) represents 50 μm.

A thin layer of the ECM represents a mature and normal muscle fibre condition (UD panels in Figure 4A,B). We profiled the distribution of ECM in damaged muscle by examining two major components in skeletal muscle: collagen IV and laminin. Following muscle damage, we detected an abnormal distribution of both markers in the CA samples on day 5 (Figure 4A,B). Here, they manifested through abnormal thickness (as compared to UD), as well as a disruption in their expression (Figure 4A,B,D,E). We noted the presence of many fibres where both collagen IV and laminin did not fully surround the muscle fibre (indicated by yellow arrows) in Figure 4A,B. These features did not manifest in the CA/sAct cohort. At later time points, both the CA and CA/sAct cohorts displayed similar collagen IV and laminin profiles.

Next, we examined the distribution of dystrophin, a key protein required to maintain muscle fibre integrity. Following muscle damage, dystrophin is lost and then returned to its normal profile (a thin layer around the inner surface of the sarcolemma, shown in UD panels in Figure 4C). Determining the extent of dystrophin expression on the inner surface (circularity) was used as a measure of fibre maturation (Figure 4F). On day 5, CA muscles showed no regenerating fibres (identified by the location of CLN) with the presence of dystrophin. However, CA/sAct contained many newly formed fibres expressing dystrophin. Nevertheless, they did not display an expression domain around the entire inner surface (only approximately 55%). By days 10 and 15, both the CA and CA/sAct cohorts showed 100% dystrophin circularity in newly formed fibres (Figure 4C,F).

### 2.5. sActRIIB Treatment Reduced Intramuscular Bleeding Following Venom-Induced Damage

**Figure 5:**
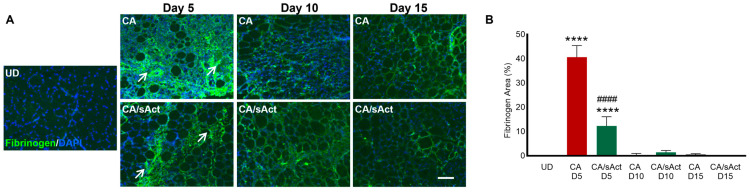
**Intramuscular bleeding in venom-damaged muscle.** (**A**) Intramuscular bleeding as indicated through fibrinogen distribution in UD, CA, and CA/sAct muscle on days 5, 10, and 15. White arrows show prominent presence of fibrinogen. (**B**) Quantification of fibrinogen coverage in damaged regions. Data represent mean ± S.D. (*n* = 4 mice in each cohort). * represents a comparison between UD muscle with either CA or CA/sAct, and # represents a comparison between CA and CA/sAct cohorts at identical times. Statistical analysis was performed using one-way ANOVA followed by Tukey’s post-hoc test. **** or #### *p* < 0.0001. The scale bar for all images represents 100 μm.

Excessive fibrinogen in the intramuscular space indicates bleeding in the muscles. The CA muscles showed high levels of fibrinogen (covering approximately 40% of damaged regions) on day 5. However, the sActRIIB treatment significantly reduced fibrinogen levels, to account for 10% coverage on day 5, indicating a potential decrease in bleeding (Figure 5A,B). The fibrinogen was largely absent in both cohorts on days 10 and 15.

## 3. Discussion

Researchers have explored various approaches to mitigate snakebite-induced muscle damage and enhance muscle regeneration, including mesenchymal stromal cell-based strategies, nanobodies, antivenoms, peptides, small molecule inhibitors, and monoclonal antibodies [52,53,54,55,56]. These efforts aim to prevent long-term disabilities [17]. Although significant advances have been made, leading to a better understanding of the different players involved and highlighting the complexity of developing new therapies, effective treatments are still not available in clinical settings [17,52]. Therefore, pre-clinical studies assessing novel strategies, such as sActRIIB treatment, are crucial.

Here, we used the whole *C. atrox* venom, in contrast to our previous study, which only utilised a specific PIII metalloprotease (CAMP) [24]. Another notable difference between the two studies is the duration of the investigations; here, we examined the muscle for 15 days, whereas previously, the research was limited to 10 days. Nevertheless, we have noted similar effects at comparable time points between the two studies. Notably, we report that damage was evident in both studies on day 10, which persisted in this study to day 15. Therefore, in both studies, the innate regeneration mechanisms were unable to resolve damage induced by CAMP alone or whole *C. atrox* venom. Hence, decreasing the relative amount of CAMP to total protein (as in this study) is still a very injurious reagent. Using whole venom may give us a better idea about the pathological conditions seen in *C. atrox* envenomation [57].

It was imperative to firstly establish the necessity of using mice in this research. Our team possesses extensive experience in using non-animal based studies to investigate the impact of venoms on skeletal muscle structure and function [8,58]. It was these and other studies that highlighted the limitation of muscle cultures and the necessity to use mice as an experimental model to generate meaningful results. This conclusion was reached after discovering that current routinely used in vitro protocols generate immature and irregular myotubes originating from one cell type, as opposed to the situation in mature muscle, which consists of long regular myofibres surrounded by numerous cell types (including endothelial, fibroblasts, and fibroadipogenic precursors (FAPs)) and a complex extracellular matrix, all of which have an impact on the muscle regeneration process. Hence, we believe that the use of mice in experimental models is vital to developing potential therapies for snake venom-induced muscle damage.

In the present study, sActRIIB treatment had the following effects on skeletal muscle after damage induced by *C. atrox* venom: 1. Muscles showed significant weight gain. 2. Regenerating fibre enlargement. 3. Decreased levels of fibrosis. 4. Decreased levels of early necrosis (but the newly formed fibre size was unaffected). 5. Collagen and laminin structures were better preserved. 6. Dystrophin expression was restored early. 7. Intramuscular bleeding was significantly reduced. Despite these positive changes, the muscle was not fully protected from the damaging influence of *C. atrox* venom by sActRIIB treatment, evident from the ongoing remodelling of ECM markers and absence of angiogenesis in both treated and untreated muscles. One of the striking features of sActRIIB treatment was on muscle weights following *C. atrox* venom injection. We noted that the TA muscles in the CA/sAct cohorts were heavier than CA muscles, an effect that was independent of the size of regeneration muscle fibres, which are identified by the presence centrally located nuclei or those that expressed the developmental form of MYH3. Importantly, the regeneration period (marked by the temporal extent of Desmin and MYH3 expression) was essentially identical in both cohorts. We postulate that the weight increase in muscle is due to the hypertrophic action of attenuating Activin/Myostatin signalling in the undamaged fibres of a muscle injected with *C. atrox* venom. Myostatin/Activin-mediated signalling is known to induce catabolic pathways, while at the same time inhibiting those responsible for fibre enlargement, mediated through the inhibition of Akt activity [59]. Hence, we suggest that at the stage of intervention, normal fibre growth was being held back, and that this inhibition was lifted by sActRIIB treatment. This line of thinking agrees with a huge body of work that shows that Myostatin/Activin inhibition is at play during the entire lifespan of animal models [60,61]. Although the regenerating fibres on day 15 were larger in the CA/sAct cohort compared to CA treatment alone, this change is unlikely to account for the change in total TA weights since they are still about one-third the area of normal fibres (UD group). Furthermore, we note that regenerating fibres from the CA/sAct cohort did not regain the size of undamaged fibres. This could be due to the limited time from damage to tissue collection, which may not have been extensive enough for the fibres to reach their full potential size.

Interestingly, the density of small cells expressing cytoplasmic Desmin and newly formed muscle fibres (expressing MYH3) did not respond to sActRIIB treatment. This implies that although Myostatin and Activin may be in circulation, myoblasts and fibres develop through programmes that were not regulated by these proteins. We propose that the beneficial effects of attenuating Myostatin/Activin following *C. atrox* venom injection is imparted in two phases, immediate and late. In the immediate phase, attenuating signalling through the ActRIIB receptor protects muscle fibres from damage, as demonstrated by the smaller area occupied by leaky muscle fibres and lower disruption of basal lamina components (laminin and collagen IV). Additionally, the sAct cohort contained fewer fibres with disrupted dystrophin expression compared to the untreated group. The protective function mediated by sActRIIB leading to attenuated fibre damage could be through its inhibition of pathways that lead to the stimulation of IL-6 expression, a potent mediator of muscle protein breakdown [37]. Herein, it has been previously shown that Myostatin binds its receptor, leading the activation of not only the Akt signalling pathway but also two other intracellular signalling cascades, the NFkβ and the p38 MAPK programmes [62]. The NFkβ pathway leads to the development of a positive feedback loop for the expression of Mstn, whereas the p38 pathway leads to the expression of IL-6, which would promote muscle breakdown. A large number of clinically approved anti-IL6 therapies have already been developed which could be repurposed to control inflammatory pathways following snake bite envenomation [63]. In the late phase, sAct lifts the repressive function of Myostatin and Activin on muscle fibres (having been damaged or undamaged). Damaged muscle fibres in the sAct cohort nevertheless do not reach normal size. The reason for this remains to be elucidated, but we can hypothesize possible explanations. The most prosaic reason is that these fibres simply have not been given adequate time to fully develop. Alternatively, we suggest that muscle fibres are influenced throughout the experimental period (15 days) by venom components that retard growth independently of the action of Myostatin/Activin. These notions will be investigated in the future in order to further optimize potential therapies for snake venom-induced muscle damage.

One of the most striking features of the sActRIIB intervention is the histologically improved structure of muscle, which takes place at the last time point. In particular, we note that there was considerably less fibrosis in the CA/sAct cohort compared to CA. Additionally, there was a small albeit significant increase in the size of regenerating fibres. Importantly, the improvement in fibrosis was not evident until the later time points. We propose that the results related to fibrosis can be explained by focusing on the fate of FAPs and how this is controlled by Myostatin/Activin. Recent research has shed light on these processes in the context of chronic muscle damage, as seen in several muscular dystrophies [64]. FAPs are present to support perfect regeneration by promoting cell activities, including myoblast expansion and fate [32]. However, the number of FAPs is carefully controlled so that only basal levels survive after damage resolution, in readiness for the next round of muscle damage. However, the meticulous control of FAPs becomes deregulated in scenarios of chronic muscle damage, leading to their survival and allowing them to differentiate into either fibroblasts or adipocytes [65]. Thereafter, fibroblast numbers can be expanded by signalling pathways activated by Activin/Myostatin [28]. Indeed, there seems to be a positive feedback loop that leads to the eventual formation of fibrosis. Herein, circulating Myostatin may initiate fibroblast proliferation. These then go on to express the myokines, leading to ever-increasing proliferation and subsequent differentiation into myofibroblasts (marked by the expression of α smooth muscle actin) [43]. sActRIIB could limit fibrosis after *C. atrox* venom damage by simply attenuating Myostatin signalling so that fibroblast expansion is limited. However, our data show that fibrosis actually develops but is then subsequently decreased. We propose a new role for Activin/Myostatin in the development of fibrosis. We suggest that Activin/Myostatin are continually required for the formation of myofibroblasts and that in their absence, fibroblasts lose their ability to form an aberrant ECM. Instead, they revert to fibroblasts that remodel the ECM back towards a normal state. Presently, the identification of FAPs, for example through the expression of PDGRA, would not be informative, as in both untreated and treated animals, we see the development of cells capable of forming fibrosis. Instead, we need to determine the gene expression profiles of these cells informing on their ability to remodel ECMs. Hence, an approach such as single-cell spatial transcriptomics would be the appropriate platform, supporting the identification of FAP progeny, as well as their ability to change ECMs. Indeed, the ability of fibroblasts to remodel ECMs has been extensively studied, especially in the context of lung fibrosis [66]. This line of investigation is beyond the remit of the present study and requires future studies in the context of muscle to determine how fibrosis can be eliminated through the inhibition of Activin/Myostatin.

In summary, we show that attenuating Myostatin/Activin signalling improves muscle regeneration after *C. atrox* venom damage. However, the muscle was by no means completely regenerated, as evidenced by the finding that regenerating fibres failed to reach their normal size. As previously stated, this could be due to the presence of long-acting metalloprotease activity, which continually induces injury. Hence, we propose that, as well as using reagents that promote regeneration (here sActRIIB), a second line of intervention based on inhibiting key harmful molecules in venom would be advisable. With regards to the use of sActRIIB, we are mindful of hurdles in terms of cost and logistics, which must be considered, especially when using therapeutics in developing countries. Presently, we estimate that, using the doses in our animal studies, treatments for humans (based on 75 kg weight) would require in the order of 3 g of sActRIIB. Current estimates for the generation of this amount of sActRIIB come to just over $4000, and as such would be unaffordable to most in developing countries. However, we believe that the costs could be reduced by 10-fold using large-scale production processes. The molecule, due to its design, is relatively stable on the shelf (it remains active for at least 2 weeks in a fridge). In our opinion, these still represent significant impediments to the current use of sActRIIB as a treatment for SBE. However, there is huge activity in developing more cost-effective and potent molecules with greater stability that act to deliver the same outcomes, in particular those based on ligand-neutralising antibodies [67]. These may become suitable alternatives in the future. With regards to the second arm of the intervention, this involves antivenom or small molecule inhibitors [68]. We note that there are presently several approved antivenoms being used in cases of rattlesnake envenomation, including ANAVIP and CroFab [69]. Although these are very effective for treating systemic effects, there are reports of persistent cellular damage even after their use [3]. Hence, it would be intriguing to test their efficacy in preventing muscle damage when antivenom therapy is combined with regeneration-promoting agents like sActRIIB.

## 4. Materials and Methods

### 4.1. Materials

Lyophilised *C. atrox* venom and all other chemicals were purchased from Sigma Aldrich, Poole, UK, unless otherwise stated.

### 4.2. Ethical Statement

All animal experiments were conducted following the regulations and principles of the British Home Office for the Animals (Scientific Procedures) Act 1986. All procedures used in this study were reviewed and approved by the University of Reading Animal Welfare and Ethics Review Board and British Home Office.

### 4.3. Enzymatic Assays

The metalloprotease activity of *C. atrox* venom was determined using DQ^TM^-gelatin (ThermoFisher Scientific, Loughborough, UK), a fluorogenic substrate. DQ^TM^-gelatin is a substrate for collagenolytic enzymes. Briefly, the whole venom (10 μg/mL) in phosphate-buffered saline (PBS, pH 7.4) was mixed with DQ^TM^-gelatin (20 μg/mL) and incubated at 37 °C. The fluorescence levels were then measured by spectrofluorimetry continuously for 90 min (FLUOstar OPTIMA, BMG Labtech, Ortenberg, Germany) with an excitation wavelength of 485 nm and an emission wavelength of 520 nm.

### 4.4. TA Muscle Damage in Mice

A murine model of muscle damage was used to test the effects of *C. atrox* venom on skeletal muscle and the impact of subsequent treatment. C57BL/6 male mice (10 weeks old) were obtained from Charles River, UK. Mice were anaesthetised using 3.5% (*v*/*v*) isoflurane in oxygen and maintained at 2% during the intra-muscular injection procedure. The right TA muscle of mice was injected intramuscularly at a venom dose of 0.25 μg/g body weight. The venom dose was selected based on the enzymatic activity and our previous research experience with this venom. Thereafter, specific cohorts of 4 mice were given 10 mg/kg sActRIIB through the intraperitoneal route one hour after the venom injection and thereafter every third day. Mice were sacrificed by carbon dioxide inhalation, and death was confirmed by cervical dislocation. Left non-injected TA muscles from venom-injected cohort, right TA venom-injected, and right TA venom-injected muscles from sActRIIB animals were collected from mice on days 5, 10, and 15.

### 4.5. Dissection and Tissue Processing

TA muscles of mice were dissected intact and immediately frozen on isopentane cooled with liquid nitrogen. Dissected muscles were placed in pre-cooled tubes and stored at −80 °C. Muscles were mounted in an Optimal Cutting Temperature (OCT) compound and cut at 15 μm-thick transverse sections using a cryo-microtome for further analysis.

### 4.6. H&E Staining

TA muscle sections were removed from the −80 °C freezer and kept at room temperature for 15 min. Muscle sections were then rehydrated with PBS after which they were immersed in Harris haematoxylin for 2 min before washing under tap water for 2 min. Next, the sections were immersed twice in 70% acidic alcohol [70% (*v*/*v*) ethanol and 0.1% (*v*/*v*) HCl] to remove any background stain. After this, the slides were washed under running tap water for 5 min. Thereafter, the sections were immersed in 1% (*w*/*w*) eosin for 2 min before being dehydrated through an ethanol series (70%, 90%, and 100%). Finally, the slides were immersed twice in Xylene for 3 min to displace the ethanol. The slides were mounted using the DPX (Dysterine, Plastisizer, and Xylene) mounting media. The size of regenerated fibres was calculated by taking the area of 50 fibres that displayed centrally located nuclei from each section, which were then averaged.

### 4.7. Picrosirius Red Staining

Picrosirius red was used to identify fibrotic tissues by immersing sections in a heated Bouin solution (56 °C) for 15 min. Afterwards, the slides were rinsed with distilled water for 15 min at room temperature. The slides were then transferred to a jar containing picrosirius red stain and incubated for 1 h in the dark. The slides were then rinsed in two separate jars containing acidified water [0.5% (*v*/*v*) glacial acetic acid] and dehydrated three times in 100% ethanol for 5 min. Finally, the slides were cleaned by immersion in xylene for 5 min and then mounted with DPX mounting media. The fibrosis analysis was carried out using threshold analysis. A set area (200 μm × 200 μm) was selected from the undamaged muscle and a baseline set using the ImageJ (version 1.52a) threshold analysis tool. This value was compared to that from a 200 μm × 200 μm area of a damaged muscle region to give a relative value (%) of picrosirius red coverage.

### 4.8. Immunohistochemistry

The sections were rehydrated by washing with PBS, followed by 15 min of incubation with a permeabilization buffer [20 mM HEPES, 3 mM MgCl_2_, 50 mM NaCl, 0.05% (*w*/*v*) Sodium Azide, 300 mM sucrose, and 0.5% (*v*/*v*) Triton X-100]. After washing with PBS, non-specific binding of antibodies was prevented using a blocking wash buffer [PBS with 5% (*v*/*v*) foetal bovine serum and 0.05% (*v*/*v*) Triton X-100] and incubating for 30 min at room temperature. Premade primary antibodies (details are provided in supplementary information) in blocking wash buffer were incubated with the sections overnight at 4 °C. Unbound primary antibodies were then removed by washing the sections with a blocking wash buffer three times. Next, the sections were incubated with secondary antibodies (diluted in blocking wash buffer) for 1 h in the dark at room temperature. The slides were then mounted with a fluorescent mounting medium containing 4,6-diamidino-2-phenylindole (DAPI) (Fisher Scientific, Loughborough, UK) with Dako fluorescence mounting medium (Dako, Poole, UK). The muscle sections were visualised using a Zeiss AxioImager fluorescence microscope.

Analysis of immuno-stained sections was carried out as follows: The size of IgG-infiltrated or embryonic myosin MYH3-expressing fibres was determined by measuring either the percentage of fibres in an area of 200 μm × 200 μm or the area of 30 fibres from one section of each mouse using ImageJ. The measured area was compared between sActRIIB-treated and -untreated muscle fibres at the same time point to analyse the effect of the given treatment. Thickness of collagen IV and laminin were measured using ImageJ. Lines were drawn from the centre of the fibre to edge of the expression domain. The measuring tool within ImageJ was then used to ascertain the extent of the expression domain for collagen IV or laminin. A total of 30 fibres were measured from sections of each animal. Dystrophin circularity was assessed by determining the percentage of a fibre circumference that expressed dystrophin in muscle fibres with centrally located nuclei. A total of 30 centrally located fibres from one section of each animal were measured using ImageJ. The intramuscular bleeding was calculated using threshold analysis sections stained for fibrinogen using ImageJ. An area of 200 μm × 200 μm was selected from the damaged region of the muscles and compared to the baseline threshold value from undamaged muscle.

### 4.9. Statistical Analysis

Graphics for the figures were made through a commercial licence using Biorender Scientific Image and Illustration Software (https://www.biorender.com/, accessed on 21 January 2025). The collected images were processed and analysed using ImageJ and Zeiss AxioImager (4.9.1) software. Statistical analysis was performed using either an unpaired *t*-test to compare the treated and untreated groups at various time points or one-way ANOVA multiple comparison followed by Tukey’s test to compare all treated and untreated groups at various time points with UD muscle. The statistical analysis was performed using GraphPad Prism (version 7).

## Data Availability

The original contributions presented in this study are included in the article/Supplementary Materials. Further inquiries can be directed to the corresponding authors.

## Supplementary Materials

The following supporting information can be downloaded at https://www.mdpi.com/article/10.3390/toxins17020059/s1: Figure S1: Histological analysis of muscle regeneration showing infiltrating cells (A) and H&E staining for TA muscle sections on UD and CA and CA with sActRIIB on days 5, 10, and 15. Blue arrows show muscle fibres with centrally located nuclei. Black arrows show infiltrating cells. The scale bar for all images represents 100 μm.
