# Supplementary material for: Soluble Activin Receptor Type IIB Improves Muscle Regeneration Following Crotalus atrox Venom-Induced Damage
Source: Toxins (Basel). 2025 Jan 28;17(2):59. doi: 10.3390/toxins17020059 (PMC11861606; doi:10.3390/toxins17020059)
Supplement: Supplementary file 1 [file toxins-17-00059-s001.zip › toxins-3310125-supplementary.pdf]

Supplementary Materials: Soluble Activin Receptor Type IIB-Mediated Inhibition Improves Muscle Regeneration Following *Crotalus atrox* Venom-Induced Damage

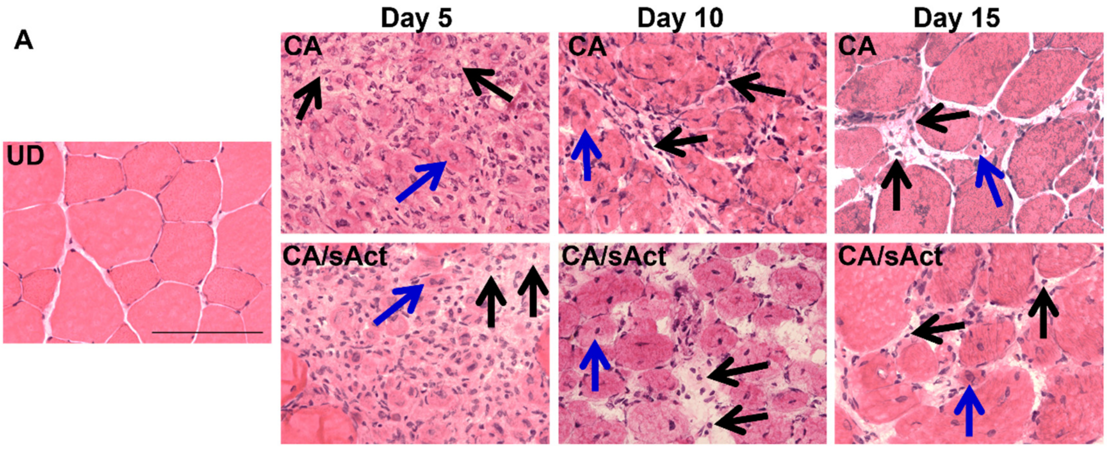

Supplementary Figure S1: Histological analysis of muscle regeneration showing infiltrating cells (A) H&E staining for TA muscle section on UD and CA and CA with sActRIIB at days 5, 10 and 15. Blue arrows show muscle fibres with centrally located nuclei. Black arrows show infiltrating cells. The scale bar for all images represents 100μm.
